# Experimental and Numerical Investigation of the Dynamic Response of a Self-Adhesive Stiffened Polyimide Foam-Based Sandwich Structure Under Blast Loading

**DOI:** 10.3390/polym18151797

**Published:** 2026-07-23

**Authors:** Yaru Sun, Chengyuan An, Bo Cheng, Yan Liu

**Affiliations:** 1 State Key Laboratory of Explosion Science and Safety Protection, Beijing Institute of Technology, Beijing 100081, China; 2 State Key Laboratory of Advanced Power Transmission Technology, China Electric Power Research Institute, Beijing 100081, China

**Keywords:** self-adhesive foam, stiffened polyimide foam, thermal stability, blast resistance, damage mechanism

## Abstract

Polymer-based sandwich structures have garnered significant interest as energy-absorbing protective materials. However, common damage modes in composite sandwich panels include matrix cracking, delamination, core crushing or core fracture, and debonding between the face sheets and the core. Among these, face–core debonding is one of the most prevalent failure mechanisms. This paper investigates a sandwich configuration designed to enhance blast resistance by incorporating a self-adhesive, stiffened polyimide foam (ASPI) into a steel–foam–steel architecture. The thermogravimetric analysis exhibits that ASPI foam obtained excellent thermal stability, and the residual mass retention at 800 °C was more than 36.2%. Experimental results show that at scaled distances of 1.077 m/kg^1/3^ and 1.292 m/kg^1/3^, the ASPI foam-based sandwich panels exhibited mid-span displacements as low as 8.5 mm and 6.7 mm, respectively. Under a scaled distance of 1.077 m/kg^1/3^, the mid-span displacement of the ASPI foam-based sandwich structure decreased from 15.0 mm to 8.5 mm, representing a 43.3% reduction compared with that of the neat polyimide foam-based sandwich structure. Moreover, compared with neat PI foam, the ASPI foam exhibited superior adhesion to steel face sheets, and no interfacial debonding was observed after blast loading. To further elucidate the underlying damage mechanisms under blast loading, a well-validated finite element model was developed and employed. Complementary scanning electron microscopy (SEM) analyses were conducted to examine the microstructural morphology of the ASPI foam core’s cross-section and surface after blast exposure. This study presents an investigation of a lightweight, self-adhesive, high-thermal stability, blast-resistant polymer-based composite foam.

## 1. Introduction

With the ever-increasing demand for enhanced safety performance in modern society, effectively improving the damage resistance and energy absorption efficiency of structures under extreme impact loading has become a critical research focus in the field of engineering materials. Across military, industrial, and civilian applications, structural materials face the dual challenge of achieving both a lightweight design and high performance. Conventional blast-resistant systems have predominantly relied on metallic materials; however, their high density and limited flexibility often fall short of practical requirements. In this context, owing to their low weight, high specific strength, and excellent energy dissipation capabilities, sandwich structures have emerged as a prominent area of research in recent years [1].

Sandwich structures typically comprise an upper face sheet, a core material, and a lower face sheet. The core is commonly composed of a lightweight substance or architecture characterized by low density and plays a pivotal role in governing the overall impact energy absorption behavior of the structure [2,3]. To improve protective performance, researchers have developed a broad range of core configurations, including metallic honeycombs, foam materials, and fiber-reinforced polymer composites [4,5]. Among these, foam materials such as metal foams [6], polyurethane foams [5], polyimide aerogel/foam [7,8,9], and polyvinyl chloride foams [10] have been widely adopted in the construction, aerospace, and transportation industries due to their low density, superior energy absorption capacity, and effective vibration damping properties. In honeycomb sandwich structures, the incorporation of a foam infill can substantially enhance energy absorption. This improvement stems from the capacity of the bubbles or pores within the foam to compress under load, thereby dissipating external impact energy. Furthermore, the internal void architecture facilitates efficient dispersion and transmission of applied loads, which helps delay premature structural deformation. Additionally, the presence of foam within the sandwich core provides supplementary lateral support, thereby increasing the overall stiffness and resistance to deformation of the structure [11,12].

In recent years, growing attention has been directed toward the dynamic response of foam-filled sandwich structures under blast loading. Cheng et al. [10] performed finite element analysis on corrugated sandwich plates filled with polyvinyl chloride foam and observed that foam filling substantially mitigates damage to the front-face sheet. Yazici et al. [13] combined experimental and numerical approaches to study the effect of foam filling on corrugated steel core sandwich plates subjected to blast loads. Their findings revealed that foam filling reduced deflections in both front and back face sheets by over 50%, with only a 2.3% increase in mass. Wu et al. [14] investigated polyurethane foam-filled auxetic honeycomb sandwich beams under blast loading and similarly demonstrated a marked improvement in blast resistance compared to unfilled counterparts. For brevity, such configurations are referred to here as foam-filled auxetic honeycomb sandwich structures. Langdon et al. [15] examined sandwich panels composed of polyvinyl chloride foam cores and glass fiber-reinforced vinyl ester face sheets under localized blast loading. In practice, achieving an optimal tradeoff between blast resistance and lightweight design is crucial for maximizing protective performance. Under blast-induced shock waves, composite sandwich panels commonly exhibit multiple failure modes, including matrix cracking, delamination, core crushing, core fracture, and face–core debonding [16]. Among these, face–core debonding is a prevalent failure mechanism in composite sandwich structures [17,18].

Many investigations [19,20,21,22] indicate that interfacial debonding acts as one of the primary failure modes of sandwich structures under dynamic loading. Although foam-filled corrugated, auxetic, and honeycomb sandwich panels exhibit favorable energy absorption performance, their performance is frequently restricted by face sheet/core debonding under high-strain-rate loading. Such interfacial failure results in degraded structural integrity and premature structural collapse. Lu et al. [19] and Russell et al. [20] employed epoxy adhesives to bond face sheets. Russell et al. [20] noted that although debonding dissipates only a small fraction of energy, it imposes a remarkable influence on dynamic deformation modes. Hassan et al. [21] revealed that upon the initiation of debonding, the core loses the ability to restrain out-of-plane deflection of the panels and ultimately suffers fragmentation. Therefore, exploring the effect of face–core bonding conditions on blast resistance is of great significance. Auxetic sandwich cores display strong deformation anisotropy; bidirectional strain-rate mismatch produces large interfacial shear displacement exceeding adhesive strength, resulting in massive interfacial debonding [22]. Honeycomb panels with low-energy-dissipation closed-cell cores concentrate impact energy at cell walls and trigger interfacial spallation [20]. Conventional polymer foams are highly brittle and cannot effectively attenuate transient blast impulses, which exacerbates interfacial debonding within foam sandwich structures [17,18]. Designing high-performance polymer foam cores with strain-rate-dependent mechanical properties mitigates interfacial debonding, since their rate-dependent stiffening response attenuates interfacial stress wave transmission and suppresses crack initiation [23,24,25]. Shear-stiffening gel is especially promising owing to its unique strain-rate-dependent behavior: its storage modulus increases sharply under high-strain-rate loading, enabling efficient absorption and dissipation of impact energy [26,27]. Our prior works [28,29] showed that shear-stiffening gel adheres strongly to fibers. Related studies have confirmed that embedding this gel within the three-dimensional interlaced architecture of fabrics can significantly enhance their impact resistance. This improvement stems from the pronounced shear-stiffening response of the gel under dynamic loading, which allows the fabric system to dissipate impact energy more effectively [30,31]. However, research on combining shear-stiffening gel with polymer foam materials to improve blast resistance remains scarce. Polyimide foam is a rigid, crosslinked, open/closed-cell foam with excellent mechanical properties and chemical stability [32]. As a core material, it can absorb substantial energy and has demonstrated outstanding performance in aerospace, marine, transportation, and mechanical engineering applications [33,34,35]. Integrating shear-stiffening gel with polyimide foam can enhance both the interfacial bonding and the mechanical strength of the polyimide matrix.

Guided by the composition–structure–function integration paradigm, this study designed and fabricated a self-adhesive stiffened polyimide (ASPI) foam-based sandwich structure. The structure was subjected to air-blast loading, and the blast resistance of ASPI cores was comparatively evaluated based on out-of-plane deformation and damage characteristics. A numerical simulation model was developed to replicate the dynamic response of the steel/ASPI/steel (S/ASPI/S) sandwich configuration. Furthermore, the energy dissipation mechanisms inherent to the engineered ASPI foam were systematically analyzed.

## 2. Experimental Section

### 2.1. Materials

The Q235 steel with dimensions of 260 × 50 × 1 mm^3^ was purchased from Beijing Junantai Protection Technology Co., Ltd. (Beijing, China). The neat polyimide (PI) foam and self-adhesive stiffed polyimide (ASPI) foam were prepared based on previous works [7,28]. The detailed synthesis process can be found in the Supplementary Material. In short, ASPI composite foam is prepared by filling polyimide foam with shear-stiffening micropowder. The density of polyimide foam adopted to fabricate ASPI is 40 kg/m^3^. Since blast-resistance performance is closely correlated with material density, higher density yields superior explosion-proof effectiveness. To facilitate a fair comparison of the protective performance of ASPI specimens, the density of neat PI foam for explosion-proof tests was adjusted to match that of ASPI via hot pressing, with both materials reaching 184 kg/m^3^. Polyurea (PUA) adhesive was prepared as in our previous work [36,37].

### 2.2. Preparation of ASPI Foam-Based Sandwich Structure

**Figure 1:**
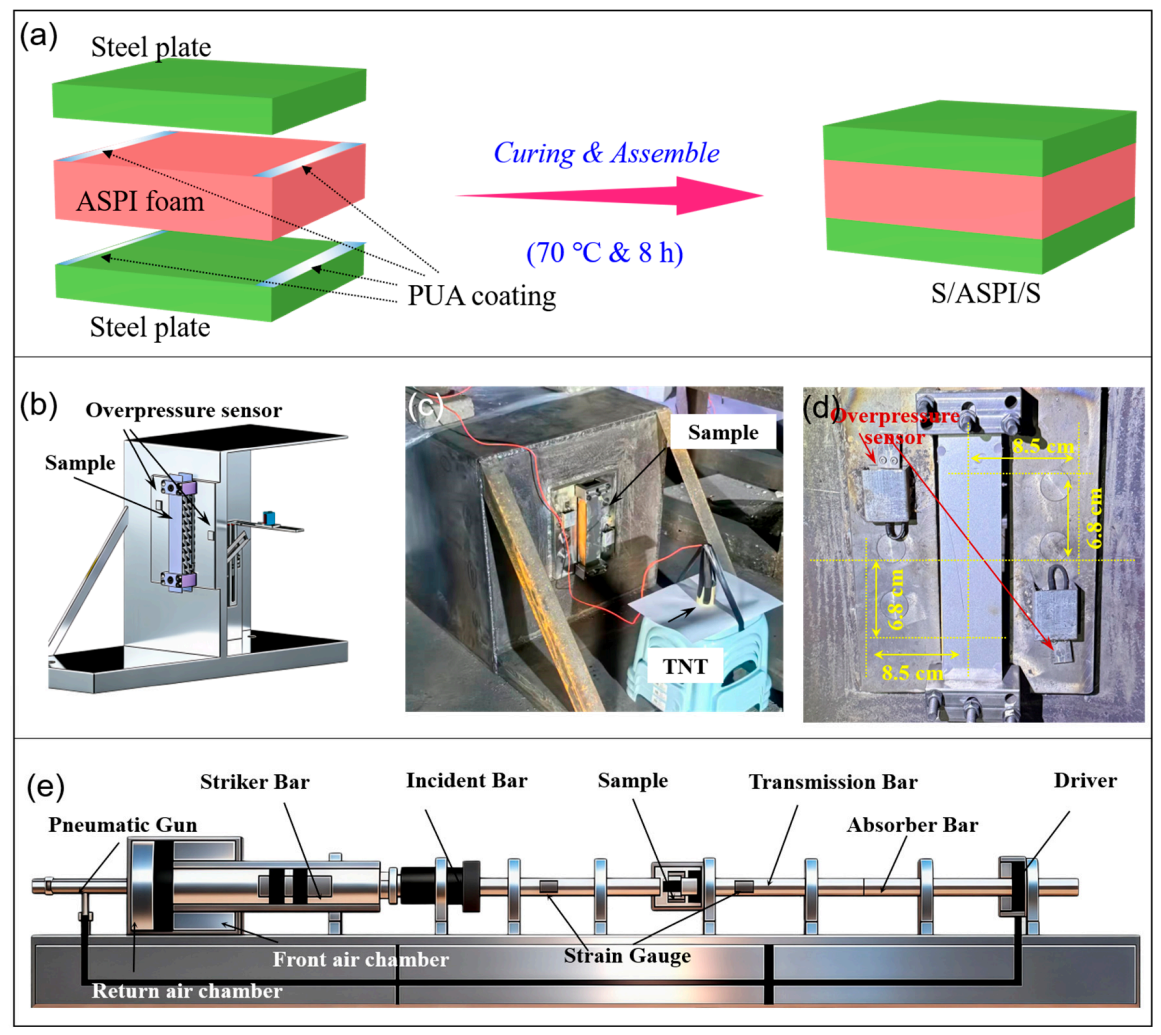
Schematic fabrication procedure of (**a**) S/ASPI/S sandwich structure; blast experimental system: (**b**) schematic illustration, (**c**) layout of the experimental site, (**d**) pressure gauges; and (**e**) schematic illustration of the SHPB experimental system.

As shown in Figure 1a, the ASPI foam-based sandwich structure (S/ASPI/S) is fabricated by bonding an SPI foam core layer to Q235 steel face sheets, followed by curing in a vacuum oven at 70 °C for 8 h. The edges of the ASPI foam are sealed to the steel face sheets using a PUA adhesive; detailed information on this adhesive can be found in our prior work [36,37]. Notably, under blast loading, the shear-stiffening micropowder filler embedded in the ASPI foam composite absorbs energy and fractures, transforming into a colloidal phase that migrates toward the interface between the face sheet and the core. In the S/ASPI/S configuration, the steel face sheets measure 260 × 50 × 1 mm^3^, while the foam core measures 200 × 50 × 30 mm^3^. Full specimen specifications are provided in Table 1. The blast stand-off distance is defined as the straight-line distance between the centroid of TNT charge and the geometric center of sandwich specimen.

### 2.3. Experimental Apparatus and Characterization Method

#### 2.3.1. Blast Test Setup

The setup for the explosion test is depicted in Figure 1b–d. S/ASPI/S sandwich structures were experimentally tested under a blast explosion induced by 0.1 kg 2,4,6-trinitrotoluene (TNT) explosive at two stand-off distances of 50 cm and 60 cm, corresponding to scaled distances Z of 1.077 m/kg^1/3^ and 1.292 m/kg^1/3^, respectively. The TNT was securely attached to the steel support using tape, with the charge positioned horizontally at a height aligned with the central point of the specimen. Furthermore, the detonation of the TNT charge was initiated directly by the detonator, which possesses an approximate TNT equivalent of 1.07 g. The S/ASPI/S sandwich structure was fixed to the support by bolts. Two pressure gauges were positioned adjacent to the beams on the solid support to evaluate the blast overpressure at a similar stand-off distance. The experimental setup incorporates the utilization of a piezoelectric sensor, specifically the KD-2013-10 model, as the pressure sensor. The KD-2013-10 piezoelectric sensor model exhibits a maximum measurement capacity of 10 MPa. Detailed information on the blasting test setup is presented in a previous work [38]. The horizontal and vertical distances from the piezoelectric sensor to the specimen center are 8.5 cm and 6.8 cm, respectively.

#### 2.3.2. Split Hopkinson Pressure Bar (SHPB) Test Setup

The compressive properties of the ASPI foam composite under impact were tested using a split Hopkinson pressure bar (SHPB), as presented in Figure 1e. Detailed information on the SHPB system is presented in previous works [36,39]. A sample with a diameter of 12.7 mm and a thickness of 2.0 mm was used for testing. The SHPB system mainly consists of a striker bar, incident bar, and transmitter bar. All the bars have a diameter of 19 mm and are fabricated from steel. In the tests, the striker bar is driven by a gas gun to strike the front end of the incident bar, generating a compressive stress wave that applies dynamic loading to the specimen sandwiched between the incident and transmitter bars. When the compressive stress wave travels to the specimen, part of the wave reflects at the interface and forms the reflected stress wave within the incident bar. The remaining portion transmits through the specimen, inducing dynamic compression, and further propagates into the transmitter bar as the transmitted stress wave. Throughout the testing process, two pairs of strain gauges are mounted on the bars to record stress wave signals. Based on one-dimensional stress wave theory, the stress and strain histories of the specimen can be derived from the measured strain signals. In this study, a stress reversal technique was implemented on the SHPB setup to prevent secondary loading of specimens induced by reflected stress waves, thereby preserving the fracture morphologies of samples.

#### 2.3.3. Material Characterization

Thermogravimetric analysis (TGA) was explored by a STA6000 simultaneous thermo-analyzer (PerkinElmer Inc., Waltham, MA, USA) under nitrogen atmosphere. Neat PI and ASPI foams (approximately 10 mg) were heated at a heating rate of 20 °C/min.

The adhesion strength values of ASPI foam and ASPI coated with PUA (ASPI-PUA) to a steel plate were measured with a universal electronic material testing machine according to previous work [37]. The dimensions of the samples were 25 × 12.5 × 2 mm^3^, and the test speed was set as 50 mm/min.

The infrared spectra of samples were investigated within the scope of 4000–400 cm^−1^ using a Nicolet IS5 Fourier transform infrared (FTIR) spectrometer (Thermo fisher Scienticfic Co., Ltd., Waltham, MA, USA).

Rheological behaviors of AASPI foam were tested using a MCR-102 rheometer (Anton Paar Co., Graz, Austria).

The micro-morphology of samples was observed from a JSM T300 scanning electron microscope (SEM) (JEOL Group, Tokyo, Japan). The samples were pre-treated by coating them with a gold layer.

A three-dimensional optical scanning device (Reeyee 2S, Nanjing Wiiboox Technology Co., Ltd., Nanjing, China) was used to further measure the morphology and damage of the samples.

## 3. Results and Discussion

### 3.1. Thermal Stability of ASPI Foam

**Figure 2:**
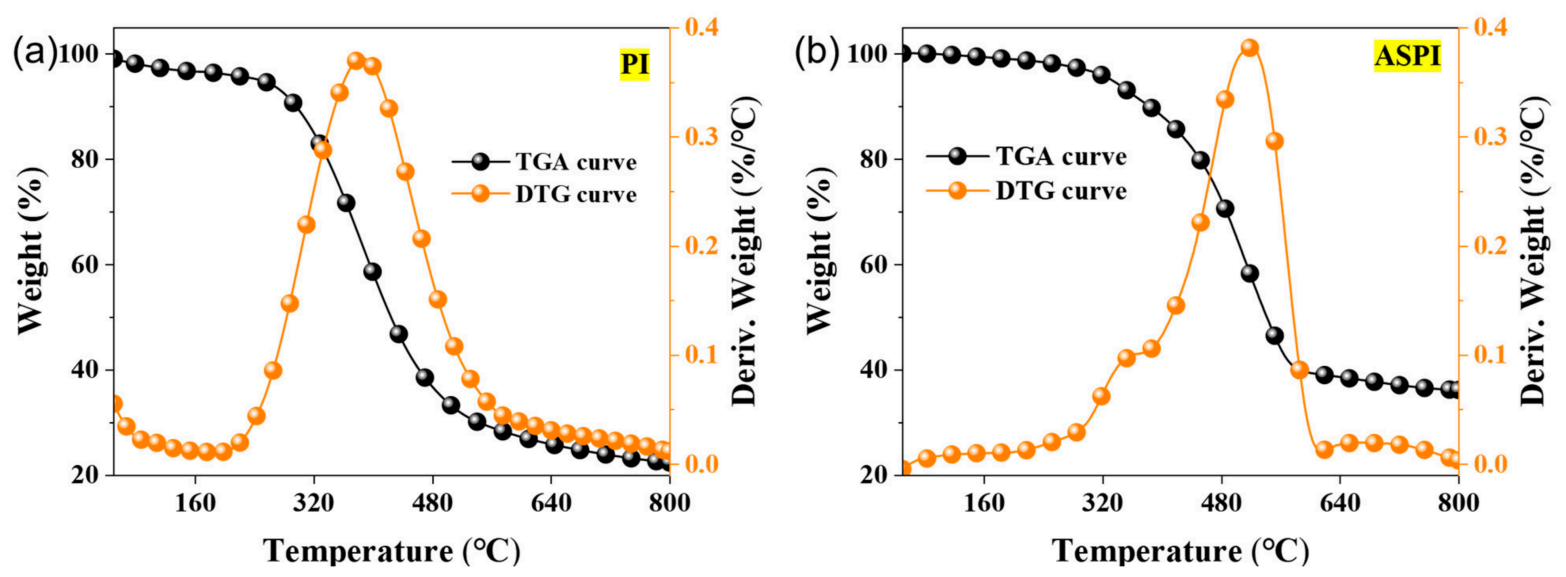
TGA curves and DTG curves of (**a**) neat PI foam and (**b**) ASPI foam.

Good thermal stability is crucial for polymer foam. Figure 2 shows the thermogravimetric analysis (TGA) and differential thermogravimetric (DTG) curves of the neat PI foam and ASPI composite foam. The maximum mass loss rate (MLR_max_), residual mass retentions at 800 °C (*R*_m_), and temperatures at 5% mass loss (*T*_5%_), and MLR_max_ (*T*d) are presented in Table 2. The TGA curve of the neat PI foam displays single-step weight loss behavior. The *T*_5%_, *T*_d_, MLR_max_, and *R*_m_ values for the ASPI foam are 330 °C, 520 °C, 0.38%/°C, and 36.2%, respectively, indicting excellent thermal stability. It is worth noting that the initial decomposition temperature *T*_5%_ of ASPI composite foam is approximately 330 °C, which is significantly higher than the composite’s preparation and processing temperature of 70 and 200 °C. This indicates excellent thermal stability during processing and confirms that the processing temperature lies within a safe range.

### 3.2. Lightweight and Adhesive Performances of ASPI Foam

**Figure 3:**
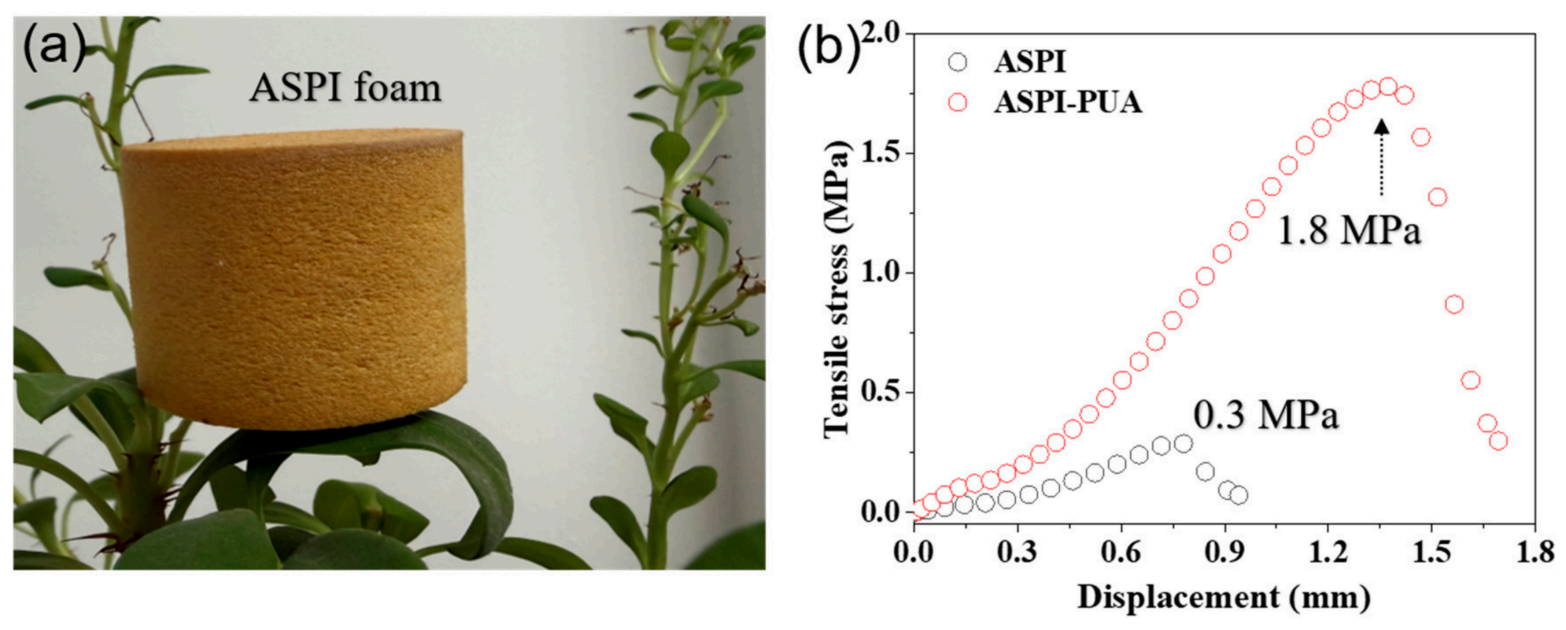
(**a**) Digital photo of ASPI, and (**b**) typical stress-displacement curve and adhesion strength of ASPI foam and ASPI-PUA.

Figure 3a shows a digital photograph of the ASPI foam composite. A cylindrical specimen rests stably on a leaf blade without deforming or fracturing the supporting stem, demonstrating its ultralow density and exceptional lightness. The schematic diagram for adhesive strength test is shown in Figure S1. The typical stress-displacement curves of the S/ASPI/S and its adhesive strength data are presented in Figure 3b. ASPI foam coated with PUA (denoted as ASPI-PUA) was bonded to steel plates using a method similar to that described in Section 2.2. Briefly, for the ASPI-PUA structure, the edges of the ASPI foam were sealed to the steel face sheets with PUA adhesive. The adhesive strength between ASPI and ASPI-PUA with Q235 steel reached 0.3 and 1.8 MPa, respectively. The ASPI foam exhibits inherent self-adhesion, and edge sealing with PUA can further improve the interfacial adhesive performance between the foam and steel substrate.

### 3.3. Mechanical Properties of ASPI Foam

#### 3.3.1. Shear Rate-Dependent Rheological Properties

**Figure 4:**
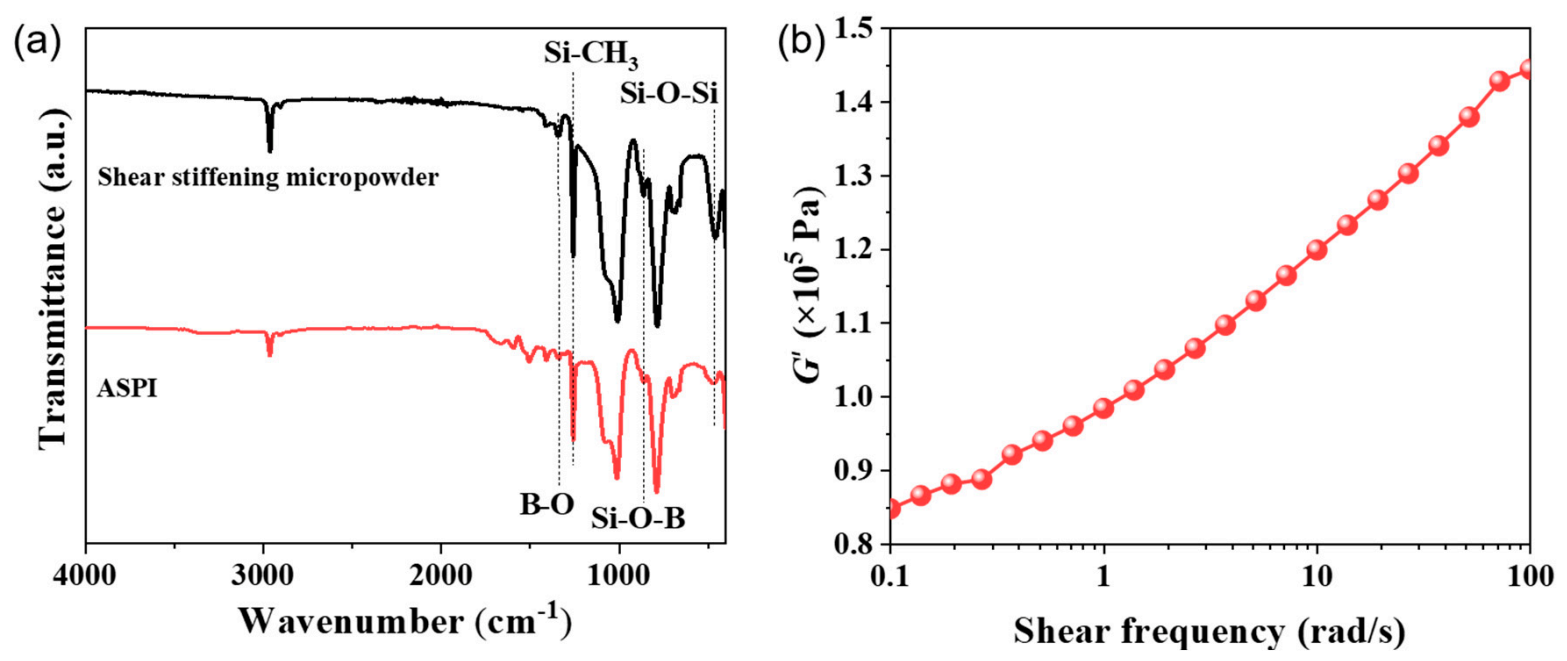
(**a**) FTIR spectra for shear-stiffening micropowder and ASPI; (**b**) *G*′ versus angular velocity curve of ASPI under rheometer test.

FTIR spectroscopy was used to identify functional groups in ASPI foam (Figure 4a). The absorption peaks at 1342 and 1260 cm^−1^ are assigned to bending vibrations of B-O and Si-CH_3_ groups, respectively. The bands at 847 and 460 cm^−1^ correspond to stretching vibrations of Si-O-B and Si-O-Si linkages [40,41,42]. Notably, the dynamic B-O coordination bonds impart shear-thickening behavior to the ASPI foam. That is, the shear stiffing micropowders enable the formation of dynamic B-O cross-linking bonds within ASPI foam. Figure 4b presents the storage modulus (*G*′) of ASPI foam obtained from shear rheological measurements. At an angular frequency of 0.1 rad/s, the ASPI foam is close to an equilibrium state owing to abundant dynamic B-O coordination bonds. As the frequency increases to 80 rad/s, the *G*′ value rises, indicating pronounced strain-rate dependence. When the frequency exceeds 80 rad/s, the *G*′ value reaches a plateau. This phenomenon arises because polymer chain relaxation occurs on a timescale shorter than the deformation period, which demonstrates the rigid internal architecture and excellent mechanical stability of the material.

Shear thickening describes the rise in apparent viscosity or modulus with an increasing shear rate in non-Newtonian systems. The relative shear-thickening effect (*RSTe*) is a dimensionless metric quantifying strain-rate sensitivity [26]. The *RSTe* is calculated according to Equation (1). A higher *RSTe* signifies greater structural reinforcement under high-rate loading. For ASPI foam, *RSTe* equals 69.4%, confirming exceptional strain-rate responsiveness.

(1) $$RSTe=\frac{G_{\max}^{\prime }-G_{\min}^{\prime }}{G_{\min}^{\prime }}\times 100\%$$

where $G_{\max}^{\prime }$ is the maximum energy storage modulus; $G_{\min}^{\prime }$ is the minimum energy storage modulus.

#### 3.3.2. Dynamic Compressive Mechanical Behavior

**Figure 5:**
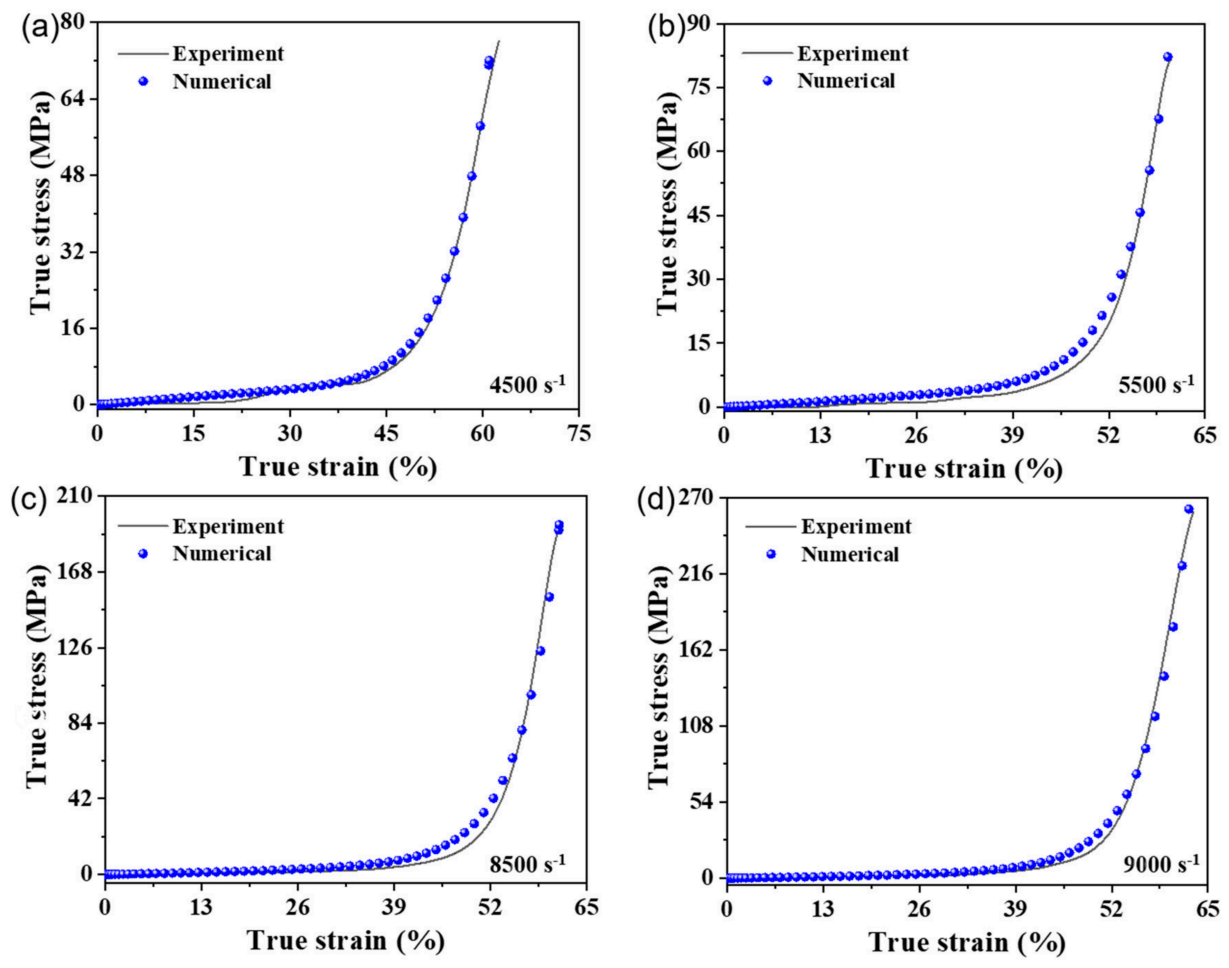
(**a**) Stress–strain curves of the ASPI foam under various compression rates: (**a**) 4500 s^−1^, (**b**) 5500 s^−1^, (**c**) 8500 s^−1^, and (**d**) 9000 s^−1^.

Figure 5 present the stress–strain curves of the ASPI foam under four high dynamic compressive strain rates: 4500, 5500, 8500, and 9000 s^−1^. The corresponding maximum stress (*σ*_max_) is exhibited in Table 3. When the compressive strain rates are increased from 4500 s^−1^ to 5500, 8500, and 9000 s^−1^, their corresponding *σ*_max_ of the ASPI composite foam is increased from 75.3 MPa to 81.7, 193.4, and 265.1 MPa. The increase in the *σ*_max_ value of the ASPI composite foam indicates that it has good strain-rate sensitivity and dynamic hardening characteristics of mechanical properties. The strain-rate sensitivity parameter (*β*), proposed by Ludwick et al. [43], was employed to quantitatively characterize the compressive strain-rate sensitivity of the material. The *β* values calculated using Equation (2) are presented in Table 3. The data show that *β* increases with increasing strain rate, indicating that the designed ASPI foam exhibits excellent impact-stiffing performance.

(2) $$\beta =\frac{\sigma_{high}-\sigma_{low}}{\log\left({\dot{\varepsilon }}_{high}\right)-\log\left({\dot{\varepsilon }}_{low}\right)}$$

where *σ*_max_ is the maximum stress, $\overset{\cdot }{\varepsilon }$ represents the strain rate, and the subscripts “*high*” and “*low*” correspond to high and low compressive loading rates, respectively. The low compressive strain rate is 4500 s^−1^, and the high compressive strain rates are 5500, 8500, and 9000 s^−1^, respectively.

#### 3.3.3. Calibration of Constitutive Parameters

The dynamic constitutive behavior of ASPI foam was characterized using a low-density foam model that accounts for strain-rate effects. Specifically, the Sherwood model [44] was employed to fit the constitutive relationship of the ASPI foam, as given by:

(3) $$\sigma =G(\rho )\cdot M(\varepsilon ,\dot{\varepsilon })\cdot f(\varepsilon )$$

where *G*(*ρ*) represents the effects of foam density on stress, while $M(\varepsilon ,\dot{\varepsilon })$ primarily characterizes the influence of strain rate on stress. The function *f* (*ε*) describes the shape of the stress–strain curve.

Since this study does not account for density effects, the influence of density on the stress–strain response is neglected. To capture the three distinct stages of the stress–strain curve, a linear function, hyperbolic tangent function, and tangent function were employed, as defined in Equation (4). The strain-rate dependence is described by Equation (5). Nonlinear parameter fitting was performed using Python’s scipy.optimize.curve_fit function, based on stress–strain data obtained at compression strain rates of 4500, 5500, 8500, and 9000 s^−1^. The resulting fitted curve is shown in Figure 5, and the corresponding parameters for each function are summarized in Table 4.

(4) $$f(\varepsilon )=a_{1}\cdot \varepsilon +a_{2}\left(e^{a_{3}\cdot \varepsilon }-1\right)+a_{4}\cdot \tan\left(a_{5}\cdot \varepsilon \right)$$

(5) $$M(\varepsilon ,\dot{\varepsilon })=b_{1}{\left(1+\frac{\dot{\varepsilon }}{{\dot{\varepsilon }}_{0}}\right)}^{b_{2}+b_{3}\varepsilon }$$

Once all parameters were determined, the model was used to predict the compressive stress–strain curves of ASPI foam over the strain-rate range of 4500–9000 s^−1^. These predictions provide essential material input data for modeling ASPI foam cores in sandwich structures.

### 3.4. Numerical Simulation Analysis

#### 3.4.1. Numerical Model

**Figure 6:**
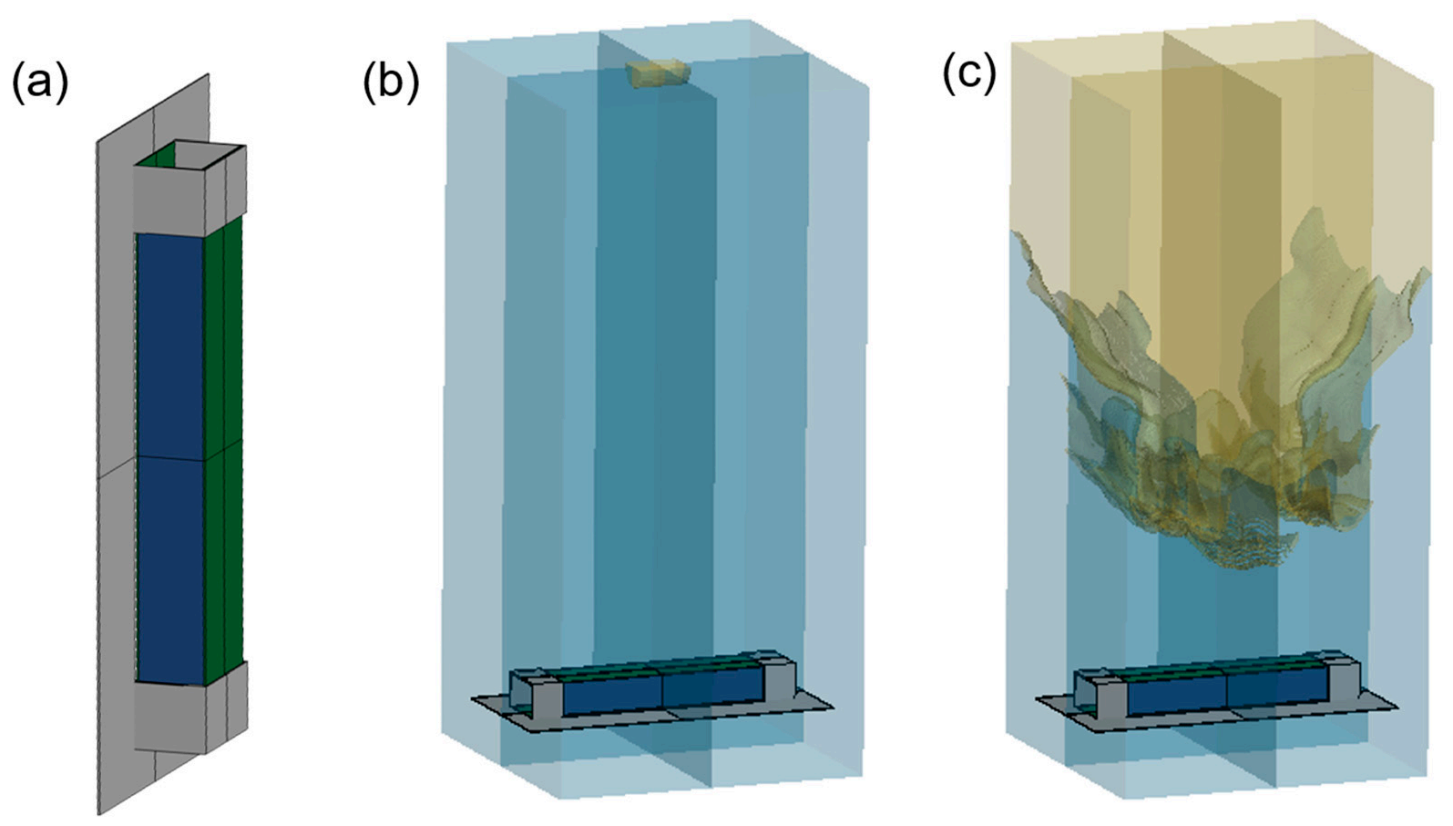
The finite element model for S/ASPI/S under blast loading: (**a**) S/ASPI/S foam model, (**b**) arbitrary Lagrangian–Eulerian, and (**c**) cross-section of the structure.

Due to the challenges associated with capturing the dynamic deformation of sandwich structures in real time during blast impact experiments, numerical simulation has emerged as an effective alternative approach [45,46]. Finite element analyses were conducted using LS-DYNA, and the complete computational model is illustrated in Figure 6.

The air domain and TNT charge are discretized using Arbitrary Lagrangian–Eulerian (ALE) meshes, generated parametrically via the S-ALE formulation. To improve computational efficiency, a quarter-symmetry model is employed for the sandwich specimen, while the TNT charge is modeled with eighth-symmetry. The outer boundary of the air domain is assigned non-reflecting symmetry conditions to approximate an infinite spatial domain. The TNT explosive is initialized using the *INITIAL_VOLUME_FRACTION_GEOMETRY keyword. Both the specimen and the fixtures are modeled with Lagrangian hexahedral elements. Specifically, the steel plate is meshed with a resolution of 0.3 mm in the thickness direction (Z-direction) and 1 mm in the in-plane directions (X and Y); the foam core and ALE regions uniformly employ a 1 mm element size. Regarding boundary conditions, reflective symmetry constraints are applied on the symmetry planes using *BOUNDARY_SPC_SET. The fixture is fully fixed (all six degrees of freedom constrained), and translational displacements in the X- and Y-directions are restricted at the bolt–hole interfaces between the fixture and steel plate to replicate actual assembly constraints. For coupling and contact definitions, fluid–structure interaction between the Lagrangian solid structure and the ALE fluid domain is modeled using *CONSTRAINED_LAGRANGE_IN_SOLID. The interface between the steel plate and the ASPI foam core is tied using *CONTACT_TIED to enforce a fully bonded, no-slip condition.

#### 3.4.2. Material Model

Material parameters and relevant simulation settings are as follows. Both air and TNT have been identified by *ALE MULTI MATERIAL GROUP in the numerical model. The exploding detonation properties of TNT were controlled using the keycard *MAT_HIGH EXPLOSIVE_BURN. A JONES-WILKINS-LEE (JWL) equation establishes pressure in terms of the initial energy per initial volume (*E*) and the relative volume (*V*) in the equation of state (EOS) of TNT as follows.

Air is treated as an ideal gas, governed by the ideal gas equation of state:

(6) p=(γ−1)ρe

where the ratio of specific heats (*γ*) is 1.4, density (*ρ*) is 0.001225 g/cm^3^ and pressure cutoff (*p*_0_) is 0.101 MPa.

TNT is described using the JWL equation of state:

(7) $$p=A\left(1-\frac{\omega }{R_{1}V}\right)e^{-R_{1}V}+B\left(1-\frac{\omega }{R_{2}V}\right)e^{-R_{2}V}+\frac{\omega E}{V}$$

where *A*, *B*, *R*_1_, *R*_2_, and *ω* are the parameters of the JWL equation of state. The specific values used in the calculations are listed in Table 5. In the table, *ρ*_0_ denotes the initial density of the explosive, *p*_cj_ is the Chapman-Jouget pressure, *D*_cj_ is the detonation velocity, and *E*_0_ is the initial internal energy per unit volume of the explosive.

The Q235 steel plate is modeled using the *MAT_PLASTIC_KINEMATIC (MAT_003) in LS-DYNA. The relevant material parameters are listed in Table 6. The yield stress of the material as a function of the equivalent plastic strain and strain rate is expressed as:

(8) $$\sigma_{y}=\left[\sigma_{0}+E_{t}\varepsilon_{p}\right]\left[1+{\left(\frac{\dot{\varepsilon }}{C}\right)}^{1/P}\right]$$

where *E* is the elastic modulus, *ν* is Poisson’s ratio, *ρ* is the material density, and *σ*_y_ is the dynamic yield stress. *σ*_0_ denotes the initial yield stress, *E*_t_ is the tangent modulus after yielding; *ε*_p_ is the equivalent plastic strain; and $\overset{\cdot }{\varepsilon }$ is the equivalent plastic strain rate. *C* and *p* values are the Cowper-Symonds strain-rate parameters.

The fixtures are modeled as rigid bodies using the MAT_RIGID material model and are fully constrained in the global coordinate system. The ASPI foam core is assigned the constitutive model developed earlier (Equations (3)–(5)), in which the stress depends on both strain and strain rate. In LS-DYNA, this material is implemented using the MAT_MODIFIED_CRUSHABLE_FOAM keyword, which supports the input of stress–strain curves at multiple strain rates and automatically interpolates the response for intermediate strain rates.

#### 3.4.3. Simulation Model Validation

**Figure 7:**
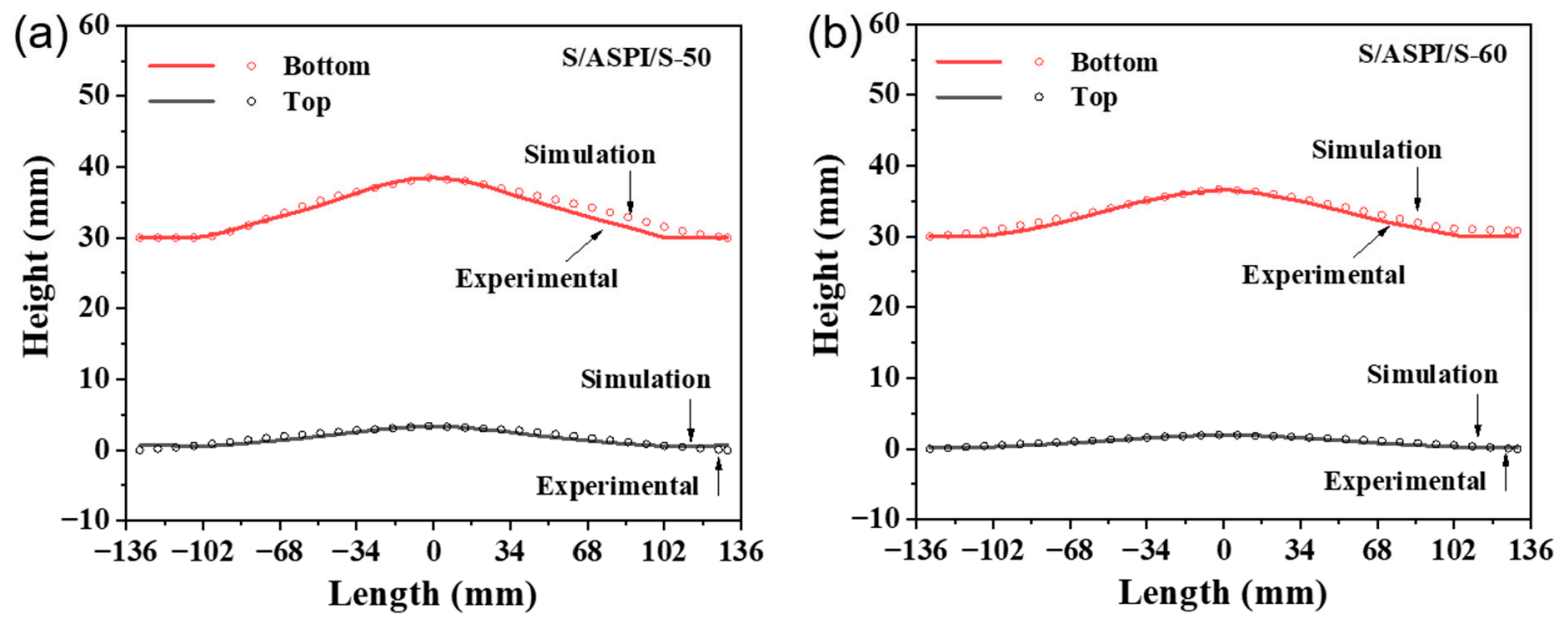
Comparisons of displacements of specimens under experimental and simulated blast conditions: (**a**) S/ASPI/S-50 and (**b**) S/ASPI/S-60.

In the simulation model, the maximum deflections of the front and back face sheets of the S/ASPI/S-50 sandwich structure are 3.6 mm and 8.3 mm, respectively, compared to the experimental values of 3.4 mm and 8.5 mm, yielding errors of only 5.9% and 2.4%. The maximum deflections of the front and back face sheets of the S/ASPI/S-60 sandwich structure are 1.8 mm and 6.6 mm, respectively, compared to the experimental values of 2.0 mm and 6.7 mm, yielding errors of only 10.0% and 1.5%. As shown in Figure 7, the overall deformation profile obtained from the simulation closely matches the experimental data. These results demonstrate the validity and accuracy of the numerical model for the S/ASPI/S structure.

### 3.5. Blast Resistance and Mechanism of the S/ASPI/S Sandwich Structure

**Figure 8:**
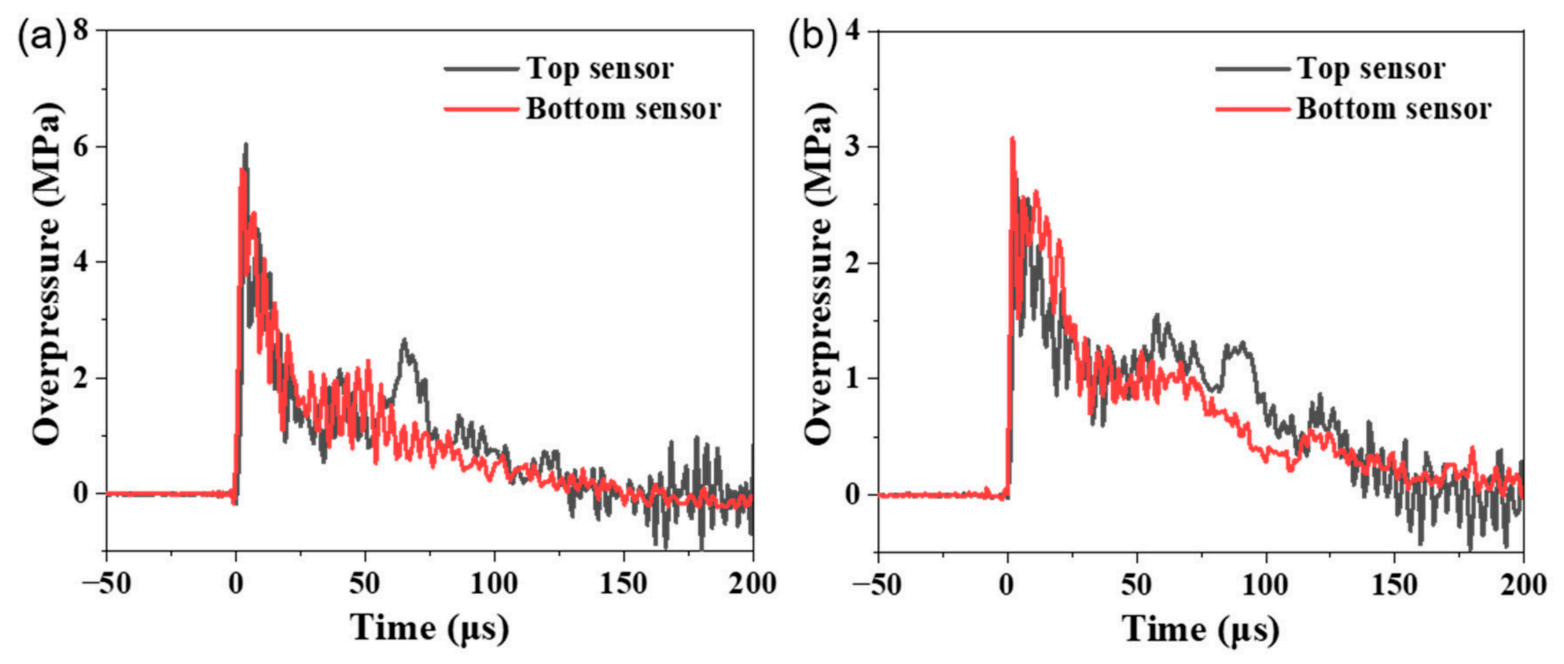
The reflected pressure histories under 0.1 kg TNT blast loading: (**a**) 50 cm (*Z* = 1.077 m/kg^1/3^) and (**b**) 60 cm (*Z* = 1.292 m/kg^1/3^).

In this section, ASPI foam was combined with a steel plate to further investigate the blast impact protection performance of a core-based sandwich structure. In general, the anti-impact performance is evaluated based on structural deformation and core-layer integrity. The results of typical reflected pressure histories are given in Figure 8. The scaled distance *Z* is defined as a function of the equivalent TNT mass M and the stand-off distance *h*_m_, expressed as Equation (9). This scaling law indicates that two explosive charges with similar geometries, identical explosive components, and different masses can produce self-similar blast waves under the same ambient conditions when their scaled distances *Z* are equal [38].

*Z* = *h*_m_/*M*^1/3^ (9)

Figure 8a,b present the time histories of reflected overpressure measured by pressure sensors. As shown in the figure, for the stand-off distance *h*_m_ = 50 cm, 60 cm, the distances from the charge to the upper and lower pressure sensors were 51.2 cm/51.2 cm and 61.0 cm/61.0 cm, respectively. Under these experimental conditions, the peak free-field overpressures recorded were 6.0 MPa/5.6 MPa and 2.9 MPa/2.8 MPa, respectively. Using the modified Kingery-Bulmash (K-B) model proposed by Shin et al. [49], which was calibrated based on near-field numerical simulations (Equation (10)), the corresponding theoretical peak overpressures were calculated as 8.5 MPa, 4.6 MPa, and 2.6 MPa. The close agreement between the experimental results and the theoretical predictions validates the effectiveness of these explosion experiments.

(10) $$\mathrm{lg}\phi =\sum_{i=0}^{n}C_{i}(K_{0}+K_{1}\mathrm{lg}{\left(h_{m}/m^{1/3}\right)}^{i})$$

where *φ* denotes the reflected peak overpressure, *m* is the mass of the TNT charge, *h*_m_ represents the stand-off distance (explosive-to-target distance), *I* is the fitting order, and *C*i, *K*0 and *K*_1_ are empirical constants determined from experimental or numerical calibration.

#### 3.5.1. Blast Resistance Performance

**Figure 9:**
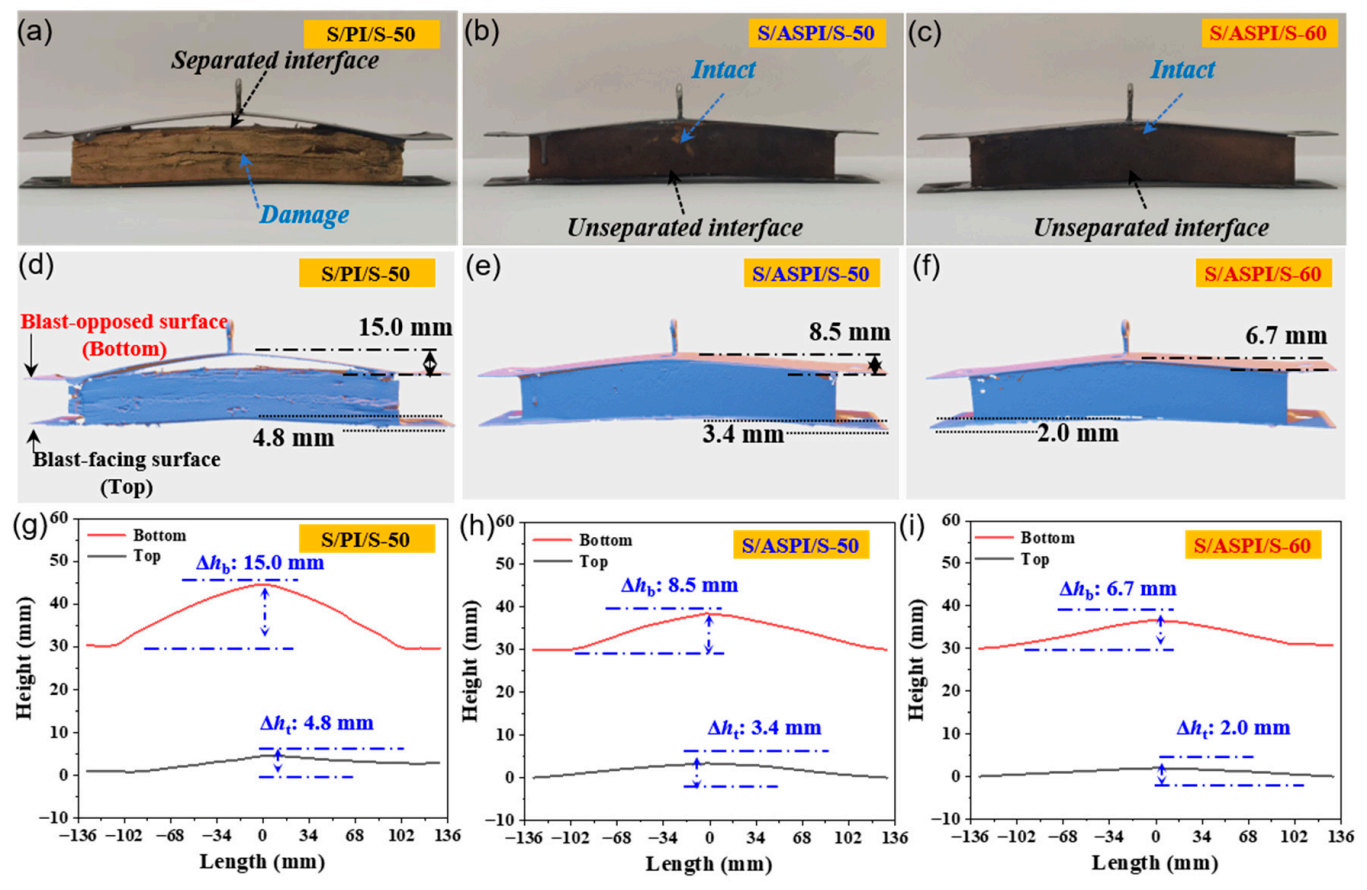
(**a**–**c**) Digital photographs of sandwich structures after the blast explosion test; (**d**–**f**) 3D scanning of S/PI/S-50, S/ASPI/S-50, and S/ASPI/S-60, and (**g**–**i**) their deformation curves of the front (**Top**) and rear panels (**Bottom**).

The digital photos of the sandwich structures before and after the blast explosion test are presented in Figure 9a–f. The typical failure modes of the S/PI/S-50 sandwich structure mainly include cracking and breaking of the core layer, as well as interface debonding between the panel and the core layer. However, no obvious damage was found in ASPI (the core material of S/ASPI/S sandwich structure). This is mainly due to the high strength of ASPI, its excellent strain-rate sensitivity effect, and its ability to withstand more blast explosive load impacts. Figure 9e,f provide a comparison of the displacements between S/ASPI/S-50 and S/ASPI/S-60. A three-dimensional optical scanning device (Reeyee 2S, Nanjing Wiiboox Technology Co., Ltd.) was used to further measure the morphology and damage of the samples. The final deformations of the bottom panel (Δ*h*_b_) of S/ASPI/S-50 and S/ASPI/S-60 are 8.5 mm and 6.7 mm, respectively, and the final deformations of the top panels (Δ*h*_t_) are 3.4 mm and 2.0 mm respectively. Under a scaled distance of 1.077 m/kg^1/3^, the Δ*h*_b_ displacement of the ASPI foam-based sandwich structure (S/ASPI/S-50) decreases from 15.0 mm to 8.5 mm, representing a 43.3% reduction compared with that of the neat PI foam-based sandwich structure (S/PI/S-50). Conventional polymer foams are highly brittle and cannot effectively attenuate transient blast impulses, which exacerbates interfacial debonding within foam sandwich structures [17,18]. In comparison, the flexible ASPI foam developed in this study maintains its complete structural morphology under blast loading without macroscopic damage, thereby preserving tight interfacial bonding between the foam core and face sheets [23,24,25].

**Figure 10:**
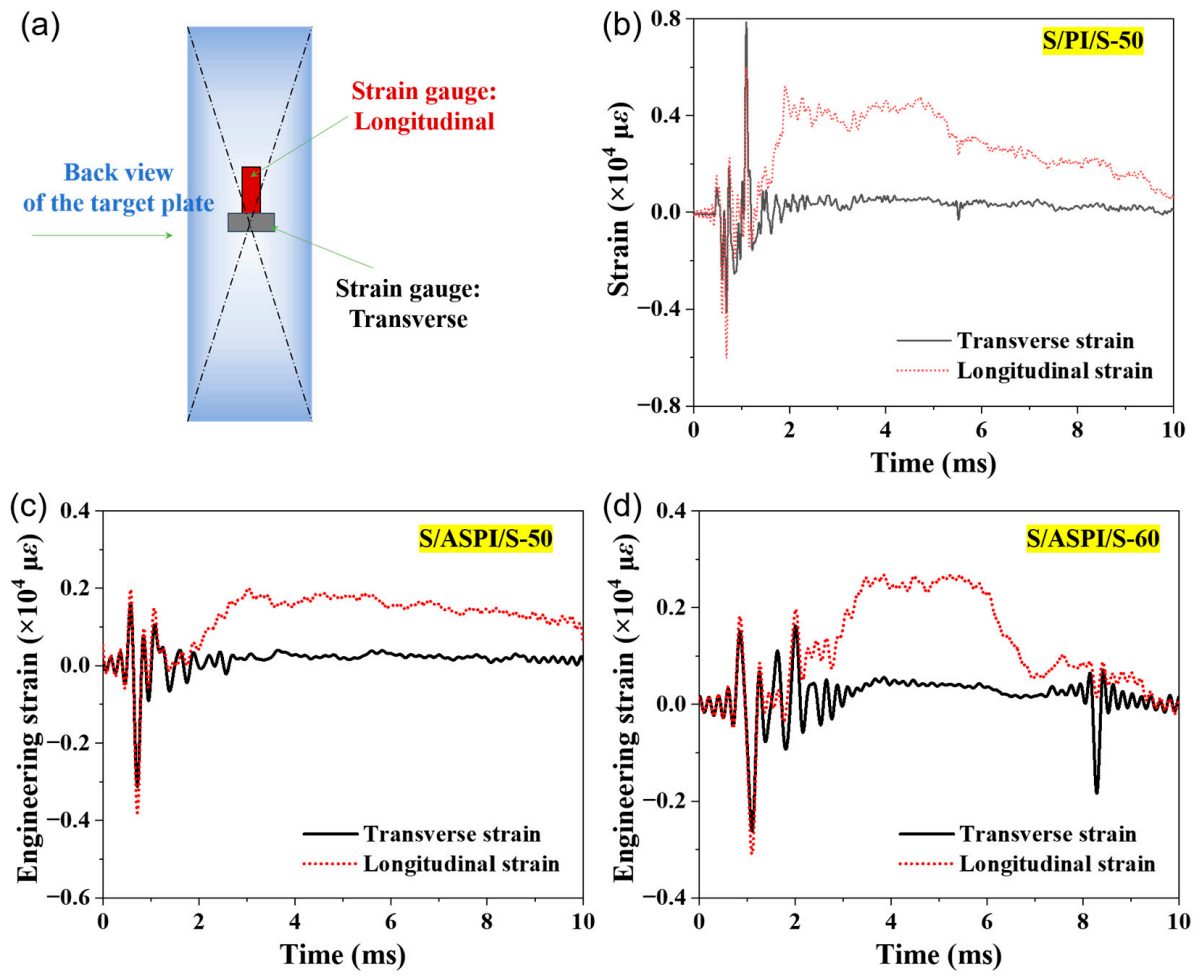
(**a**) Schematic diagram of the bonding position of the strain gauge on the back plate; strain time histories of (**b**) S/PI/S-50 (Z = 1.077 m/kg^1/3^), (**c**) S/ASPI/S-50 (Z = 1.077 m/kg^1/3^), and (**d**) S/ASPI/S-60 (Z = 1.292 m/kg^1/3^) under blast loading.

Figure 10 shows the strain time histories at the center of the sandwich specimen in the X (transverse strain) and Y (longitudinal strain) directions. The panel undergoes elastoplastic deformation immediately upon loading and subsequently settles into a relatively stable oscillatory state, during which the peak strain at the center is attained. The corresponding quantitative results are summarized in Table 7. Compared with the S/PI/S-50 specimen, the S/ASPI/S-50 specimen exhibits lower transverse and longitudinal strains, indicating that the ASPI-modified interlayer can effectively restrain the deformation of the sandwich core under dynamic loading. The following trends are observed: as the scaled distance *Z* increases, both compressive and tensile strains in the X-direction and tensile strain in the Y-direction on the back face consistently decrease. In contrast, compressive strain in the Y-direction first increases and then decreases with increasing scaled distance *Z*. At small *Z*, where the shock intensity is high and the loading rate is rapid, the back face exhibits a pronounced dynamic response. The X-direction strain is predominantly compressive due to lateral inertial confinement and the Poisson’s effect, whereas the Y-direction strain is tensile, resulting from the superposition of global bending and local stretching. As the scaled distance, *Z,* increases and the blast load attenuates, the structural response becomes milder, leading to reduced X-direction compressive strain and Y-direction tensile strain. The nonmonotonic variation of Y-direction compressive strain arises from strong shock–structure coupling at intermediate scaled distances. Stress waves undergo multiple reflections at the face sheet/core interfaces, while the core transfers load to the back face via shear mechanisms, inducing localized compressive deformation initially. When the scaled distance, *Z*, further increases and the load is insufficient to activate significant compressive response, this compressive strain diminishes accordingly.

The scaled distance, *Z*, is defined as a function of the equivalent TNT mass M and the stand-off distance *h*_m_, expressed as *Z* = *h*_m_/M^1/3^. This scaling law indicates that two explosive charges with similar geometries, identical explosive components, and different masses can produce self-similar blast waves under the same ambient conditions when their scaled distances are equal [38]. As a core normalized parameter in blast testing, the scaled distance enables standardized comparison of blast response across different experimental conditions. A comparison of the final bottom face sheet deflections (*δ*_Bottom_) between this study and literature reports is summarized in Table 8. Comprehensive comparisons of structural density and *δ*_Bottom_ distinctly demonstrate the superior protective capacity of the ASPI foam-based sandwich structure.

#### 3.5.2. Dynamic Deformation Behavior

**Figure 11:**
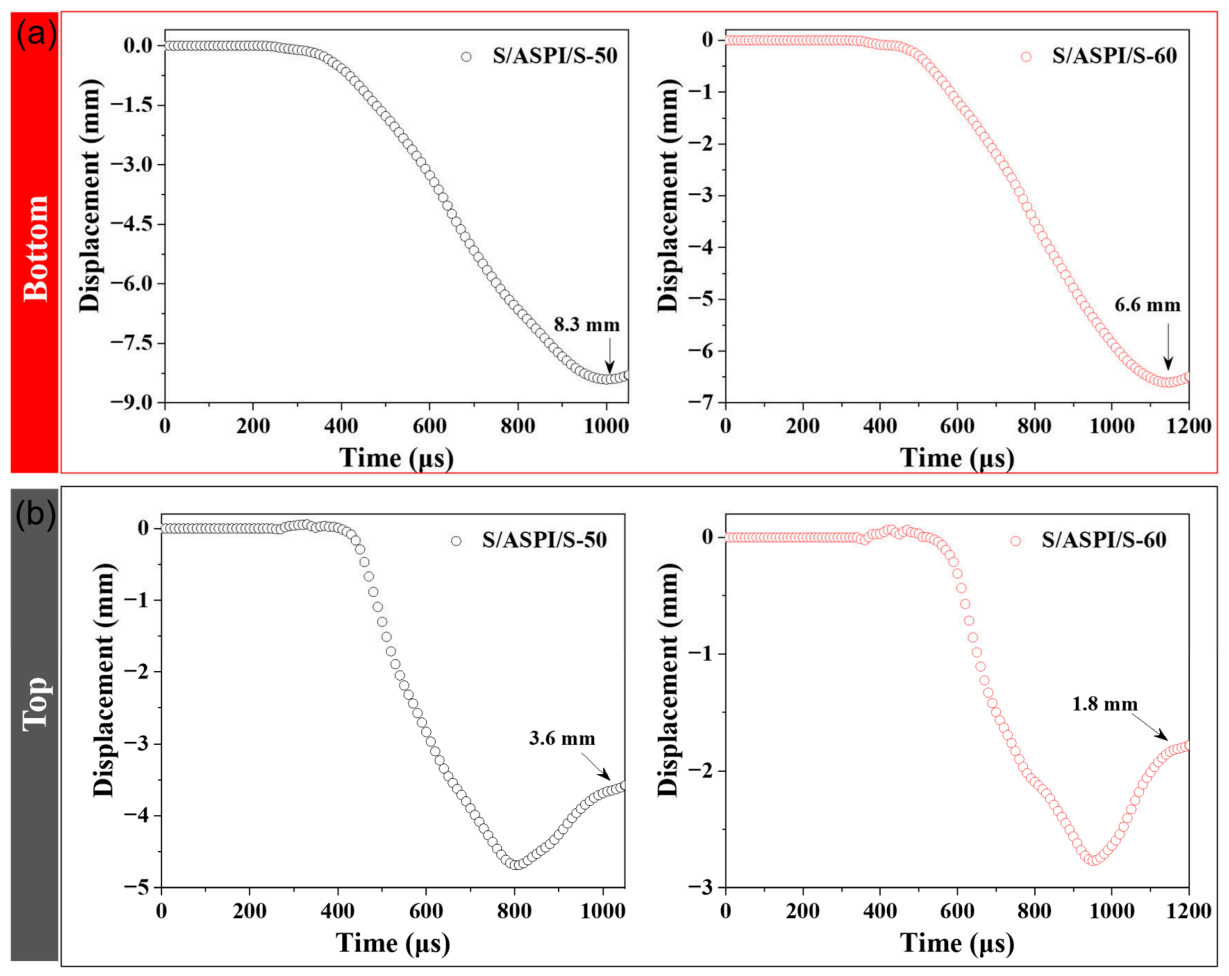
The deflection for the (**a**) bottom and (**b**) top panels in S/ASPI/S.

The above research indicates that, compared with pure PI, the sandwich structure with an ASPI composite foam as the core layer has excellent explosion impact-protection performance. To explore the dynamic response of the S/ASPI/S sandwich structure under blast loading, its deformation history was numerically simulated. The numerical simulations of a 100 g TNT explosion at scaled distances of Z = 1.077 m/kg^1/3^ and Z = 1.292 m/kg^1/3^ were conducted using LS-DYNA. The resulting time histories of the central point displacements on the front and back faces of S/ASPI/S-50 and S/ASPI/S-60 sandwich panels are shown in Figure 11.

As observed from Figure 11, the deformation of the S/ASPI/S panels gradually decreases with increasing scaled distance Z. Specifically, the maximum displacements of the back faces for S/ASPI/S-50 and S/ASPI/S-60 are 8.3 mm and 6.6 mm, respectively, exhibiting deviations of only 2.4% and 1.5% compared to physical experiments. Meanwhile, the front-face displacements are 3.6 mm and 1.8 mm for S/ASPI/S-50 and S/ASPI/S-60, respectively, with discrepancies of 2.38%, 5.9%, and 10.00% relative to experimental measurements. Nonetheless, the close agreement between the simulation and experiment further validates the accuracy of the numerical model. The displacement time histories of the back face exhibit a smooth and rapid decay, indicating efficient energy absorption by the panel system. In contrast, the front-face displacement curves show post-peak reverse oscillations. This behavior is primarily attributed to the unloading phase initiated after the external blast load dissipates, combined with the active response of the ASPI core, which releases stored energy in the reverse direction through its viscoelasticity and shear-thickening effects.

**Figure 12:**
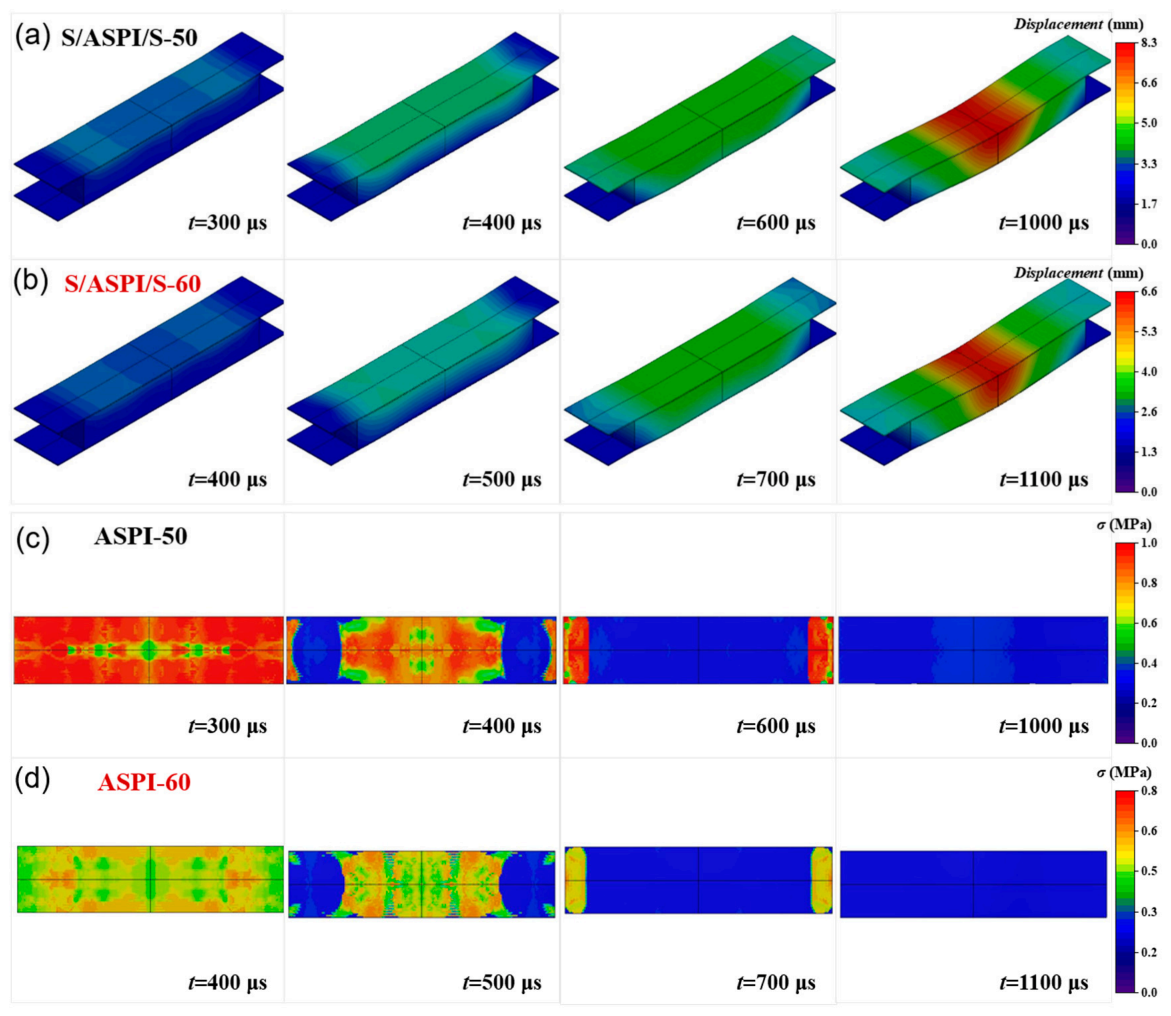
Displacement contour maps of (**a**) S/ASPI/S-50 and (**b**) S/ASPI/S-60; stress distribution contour plots in the core layers (ASPI) of (**c**) S/ASPI/S-50 and (**d**) S/ASPI/S-60.

As shown in Figure 12a,b, which presents the displacement contour map of the structure at various time instants, the deformation evolves in a distinct sequence. At 200 μs, a localized tensile deformation appears in the central region of the front-face sheet, with the displacement at the center point beginning to increase. By 300 μs, this deformed region expands radially, forming a pronounced strain concentration zone at the plate center, while the surrounding areas remain relatively undeformed, resulting in a ring-shaped strain distribution. At 500 μs, the concentrated deformation migrates toward the clamped boundaries, and significant tensile strains develop at the two corners of the back face sheet, reaching the material’s tensile yield limit and initiating plastic hinge formation. Finally, at 900 μs, the displacement field stabilizes, with the maximum residual displacement concentrated in the central region directly impacted by the blast load. This is consistent with typical failure modes observed in clamped sandwich panels under blast loading (Figure 9).

From the stress distribution contour plot (Figure 12c,d), it can be observed that as the scaled distance decreases, the stress magnitude near the blast-facing surface increases significantly, and the high-stress region becomes more concentrated. The stress wave propagation path shortens and the stress gradient steepens, indicating a stronger localized impact effect from near-field explosions. In contrast, at larger scaled distances, the stress distribution becomes more uniform, with a notably reduced peak stress and a milder overall structural response. Furthermore, stress is predominantly concentrated in the core layer (the ASPI foam), demonstrating that this layer plays a primary role in stress transmission and energy dissipation during the blast event. This effectively mitigates the loading on the face sheets and underscores the critical contribution of the core layer to the blast-resistant performance of the S/ASPI/S structure.

**Figure 13:**
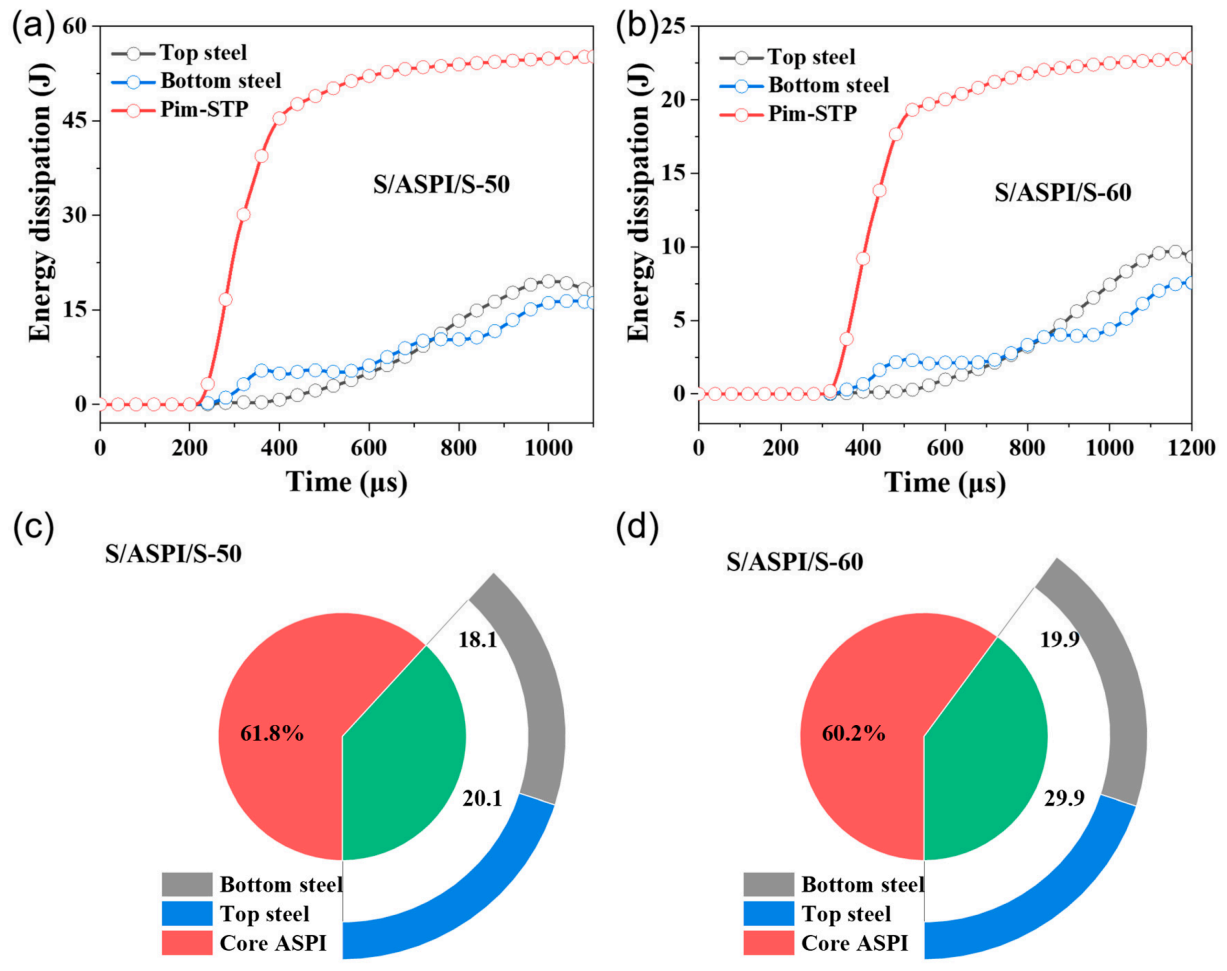
Energy absorption of each component of (**a**) S/ASPI/S-50 and (**b**) S/ASPI/-60; Total of energy absorption distribution of (**c**) S/ASPI/S-50 and (**d**) S/ASPI/-60 sandwiches.

The S/ASPI/S sandwich structure consists of front and rear steel face sheets and an ASPI foam core. Energy absorption by each component was examined at different scaled distances to reveal its blast-resistant mechanism. Figure 13a,b show that the ASPI core absorbed 57.6 J at 1.077 m/kg^1/3^ and 23.6 J at 1.292 m/kg^1/3^, indicating strong dependence on blast intensity. The foam dissipates most energy early through cell-wall buckling and densification. The front-face sheet absorbs little energy, mainly reflecting stress waves and initiating core compression. The rear face sheet contributes steadily over time during later deformation. Figure 13c,d show that as scaled distance decreases, both total energy absorption and the core’s share increase, confirming that the foam performs best under near-field blasts. At larger scaled distances, loading becomes more uniform, overall deformation reduces, and energy absorption drops, especially in the core.

#### 3.5.3. Damage Behavior of ASPI Foam

**Figure 14:**
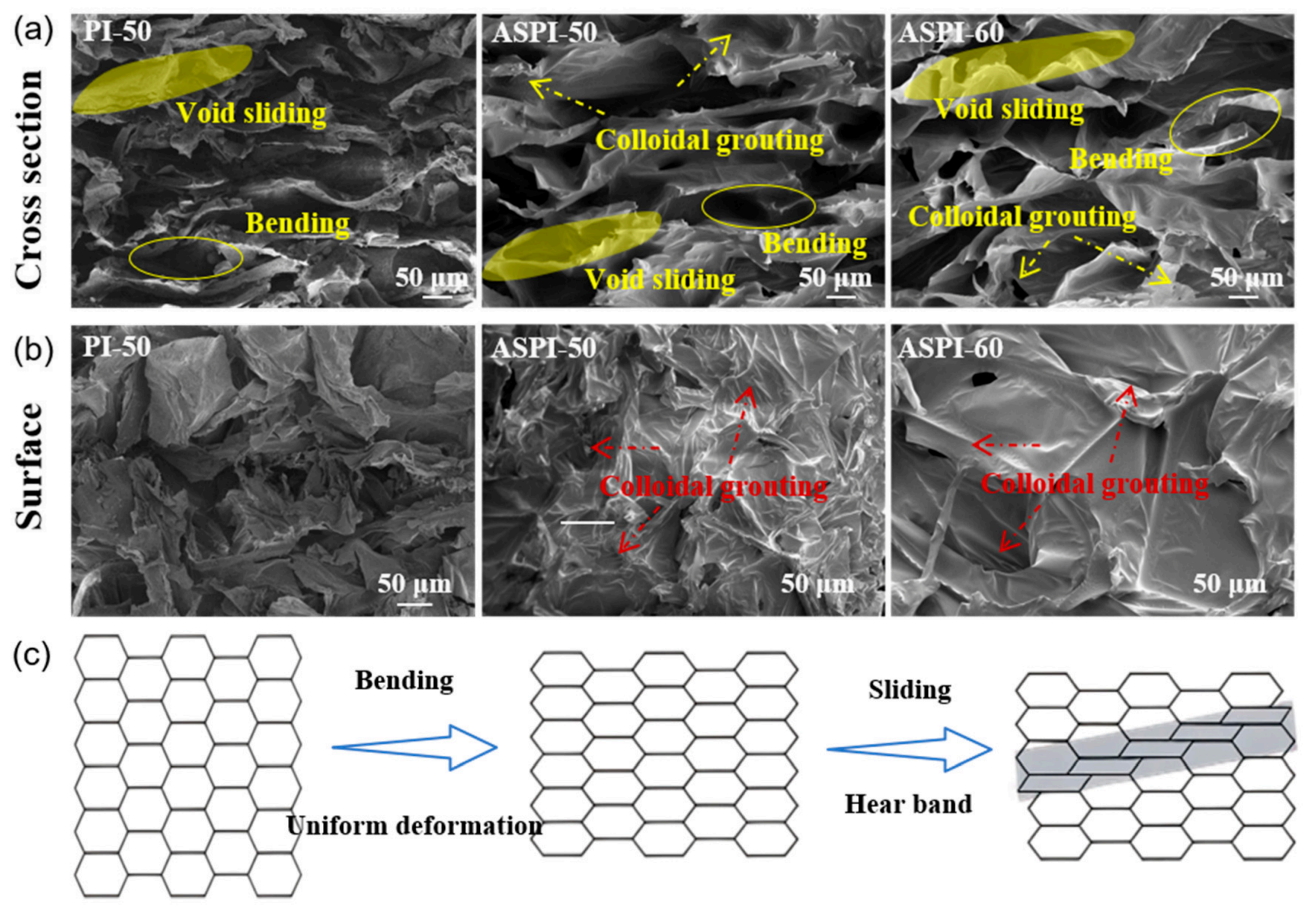
(**a**) Cross-sectional and (**b**) surface SEM images of neat PI-50, ASPI-50, and ASPI-60 foams after blast loading; (**c**) Schematic illustration of the random localized deformation mechanism in ASPI foam.

To further explore its damage mechanism, SEM was used to observe the microscopic morphology of the cross-section and surface of the core layer after the blast explosion. The results are shown in Figure 14.

As shown in Figure 14a,b, the cores of the S/PI/S sandwich structure (PI-50), as well as the S/ASPI/S sandwich structures (ASPI-50 and ASPI-60), exhibit distinct surface and cross-sectional damage after impact loading. In the SEM images, the PI-50 foam exhibits a fractured morphology. The core surface of the S/ASPI/S structure shows collapsed pores but no obvious holes. Within the ASPI foam, misalignment and shear sliding between pores lead to localized deformation bands, as illustrated in Figure 14c. The cross-section of the S/ASPI/S core retains relatively intact cell morphology, with no significant damage observed. This is primarily attributed to the excellent dynamic impact-hardening capability of ASPI foam, which effectively dissipates impact energy and suppresses bending and rotation of the cell walls.

#### 3.5.4. Mechanisms

Based on the above analysis, the sandwich structure comprising 1 mm thick steel face sheets and an ASPI foam core demonstrates excellent blast resistance under explosive loading. This performance arises from multistage shock wave propagation, attenuation, and energy dissipation within the composite system [53,54,55].

When the shock wave first strikes the front steel face sheet, the high-rate compressive stress rapidly exceeds its dynamic yield stress, triggering plastic deformation despite the material’s relatively high strength. The steel absorbs the initial kinetic energy through localized bending, micro-buckling, and plastic flow [56,57]. Concurrently, the large acoustic impedance mismatch between steel and the adjacent air or detonation products reflects a portion of the incident wave, reducing the peak pressure transmitted into the core [58]. Within the ASPI core, the shock wave undergoes amplitude attenuation and waveform dispersion. The polyimide foam exhibits pronounced strain-rate-sensitive compressive strengthening, dissipating energy via cell-wall buckling, collapse, and densification. Embedded shear-stiffening micropowders undergo an in situ transition into a gel-like shear-thickening phase under high-rate compression and shear, leading to rapid nonlinear stiffening and the formation of transient high-modulus regions. These dual strain-rate-dependent mechanisms prolong both the propagation path and the interaction time of the shock wave within the core. By the time the residual wave reaches the rear face sheet, its overpressure and impulse are substantially attenuated, effectively suppressing plastic deformation and mitigating the risk of structural failure, thereby enhancing protection of the rear space [25,59].

## 4. Conclusions

This study presents a steel–foam–steel sandwich configuration incorporating a self-adhesive stiffened polyimide foam (ASPI) to enhance blast resistance. The thermogravimetric analysis exhibits that ASPI foam obtained excellent thermal stability, with residual mass retentions at 800 °C of more than 36.2%. Experimental results demonstrate that the ASPI-based panels achieved remarkably low mid-span displacements of 8.5 mm and 6.7 mm at scaled distances of 1.077 and 1.292 m/kg^1/3^, respectively. Notably, the ASPI foam exhibited robust adhesion to the steel face sheets, with no interfacial debonding observed following blast exposure. To further understand the blast-induced damage mechanisms, a well-validated finite element model was developed, complemented by SEM analyses of the post-blast microstructural morphology of the ASPI foam core. Within the ASPI foam, misalignment and shear sliding between pores lead to localized deformation band. The cross-section of the S/ASPI/S core retains relatively intact cell morphology with no significant damage observed. This work provides the first comprehensive investigation of a lightweight, self-adhesive, polymer-based composite foam for blast mitigation, offering critical insights for the development and deployment of advanced protective sandwich structures.

## Figures and Tables

**Table 1:** Specifications of the specimens.

Sample	Stand-Off Distance (cm)	Component (Sheel/Core/Sheet)	Dimensions (mm^3^)	
			Sheel	Core
S/PI/S-50	50	Steel/PI/Steel	260 × 50 × 1	200 × 50 × 30
S/ASPI/S-50	50	Steel/ASPI/Steel	260 × 50 × 1	200 × 50 × 30
S/ASPI/S-60	60	Steel/ASPI/Steel	260 × 50 × 1	200 × 50 × 30

**Table 2:** TGA test data of neat PI foam and ASPI composite foam.

Sample	*T*_5%_ (°C)	*T*_d_ (°C)	MLR_max_ (%/°C)	*R*_m_ (%)
PI	248	384	0.37	22.4
ASPI	330	520	0.38	36.2

**Table 3:** The maximum stress (σ_max_) and strain-rate sensitivity parameter (*β*) values of ASPI foam under various compression rates.

Compression Rates (s^−1^)	*σ*_max_ (MPa)	*Β*
4500	75.3	-
5500	81.7	73.4
8500	193.4	427.6
9000	265.1	630.5

**Table 4:** The fitting coefficients of the constitutive model.

Coefficient Name	*a*_1_	*a*_2_	*a*_3_	*a*_4_	*a*_5_	*b*_1_	*b*_2_	*b*_3_
Fitted value	−0.023	0.006	0.098	0.880	0.044	5.983	−0.920	0.058

**Table 5:** JWL state equation parameters [47].

*ρ*_0_ (kg/m^3^)	*D*_cj_ (km/s)	*p*_cj_ (GPa)	*Q* (kJ/kg)	*A* (GPa)	*B* (GPa)	*R*_1_	*R*_2_	*ω*	*E*_0_ (kJ/cm^3^)
1630	6.90	21.00	4190	371.20	3.21	4.15	0.95	0.30	7.00

**Table 6:** Cowper-Symonds equation parameters [48].

*ρ* (kg/m^3^)	*E* (GPa)	*ν*	*σ*_y_ (MPa)	*E*_t_ (MPa)	*C* (s^−1^)	*p*
7850	210	0.30	235	250	40.4	5.0

**Table 7:** Peak strains on the back face of sandwich structures at different scaled distances.

Sample	Transverse Strain (με)		Longitudinal Strain (με)	
	Tension	Compression	Tension	Compression
S/PI/S-50	7863	−4155	5929	−5983
S/ASPI/S-50	1604	−3125	1997	−3806
S/ASPI/S-60	1617	−2620	1794	−3099

**Table 8:** Blast resistance results of steel sheet-based sandwich in the literature and this study.

Core Material	*Z* (m/kg^1/3^)	Core Density (kg/m^3^)	Thickness (mm)	*δ*_Bottom_ (mm)	Fracture (Y/N)	Refs
NSC	1.077	2300	70.0	53.0	Y	[50]
ACCF	1.500	270	50.0	20.0	Y	[51]
ACSF	1.500	1950	50.0	19.70	Y	[52]
PUF-filled CHSB	0.970	420	41.5	19.81	Y	[14]
CHSB	1.077	263	30.0	23.5	N	[38]
PI	1.077	184	30.0	15.0	Y	This study
ASPI	1.077	184	30.0	8.5	N	This study

Notes: *δ*_Bottom_ is the mid-span displacement of the bottom face sheet for the structure; NSC: Normal strength concrete; ACCF: Closed-cell aluminum foam; ACSF: Aluminum cenosphere syntactic foam; PUF-filled CHSB: Polyurethane-filled conventional hexagonal honeycomb sandwich beams; CHHB: Conventional hexagonal honeycomb sandwich beams.

## Data Availability

The original contributions presented in the study are included in the article/Supplementary Material, further inquiries can be directed to the corresponding authors.

## Supplementary Materials

The following supporting information can be downloaded at: https://www.mdpi.com/article/10.3390/polym18151797/s1, Figure S1. The schematic diagram for adhesive strength test.
