# Supplementary material for: Experimental and Numerical Investigation of the Dynamic Response of a Self-Adhesive Stiffened Polyimide Foam-Based Sandwich Structure Under Blast Loading
Source: Polymers (Basel). 2026 Jul 23;18(15):1797. doi: 10.3390/polym18151797 (PMC13468428; doi:10.3390/polym18151797)
Supplement: Supplementary file 1 [file polymers-18-01797-s001.zip › polymers-4427425-supplementary.pdf]

Supplementary Material

Experimental and Numerical Investigation of the Dynamic Response of a Self-Adhesive Stiffened Polyimide Foam-Based Sandwich Structure Under Blast Loading

Yaru Sun[a*], Chengyuan An[a], Bo Cheng[b], Yan Liu[a *]

[a] *State Key Laboratory of Explosion Science and Safety Protection, Beijing Institute of Technology, Beijing 100081, China*

[b] *State Key Laboratory of Advanced Power Transmission Technology, China Electric Power Research Institute, Beijing 100081, China*

* Corresponding author
E-mail: sunyr_sc@126.com (Yaru Sun); liuyan@bit.edu.cn (Yan Liu)

S1. Preparation ASPI foam

The ASPI foam employed in this study is a self-crosslinked core-shell Fe_3O_4/polyimide composite, fabricated via a three-step process.

Step 1: Synthesis of Fe_3O_4@TS-Fe. TS-Fe nanosheets were assembled through supramolecular interactions. Briefly, tannic acid (4.0 g) and Fe_3O_4 (6.0 g) were dispersed in a mixed solvent (20 mL ethanol + 40 mL water) and stirred at 80 °C for 30 min to yield Fe_3O_4@TA. A chitosan (CS) solution (60.0 g, 1.0 wt% in 1.0 wt% acetic acid) was then added and stirred for 10 min to form Fe_3O_4@TS. Subsequently, 16.0 g of $FeCl_3 \cdot 6H_2O$ aqueous solution (12.5 wt%) was slowly introduced under stirring for another 10 min, resulting in Fe_3O_4@TS-Fe precipitation. The product was collected by centrifugation, washed twice with 800 mL deionized water, and dried at 80 °C.

Step 2: Fabrication of polyimide (PI) foam. A composite foam stabilizer (10.0 g AK8805 + 10.0 g PEG600) and a catalyst mixture (8.3 g TEDA, 16.7 g DPG, 12.5 g T12, and 62.5 g TEOA) were prepared separately. These, along with polyamic acid (PAA, 20 g), deionized water (2.4 g), and Fe_3O_4@TS-Fe (4.0 g), were homogenized to form a masterbatch. Polymethylene polyphenyl isocyanate (29.6 g) was then rapidly added, and the mixture was stirred at 2000 rpm for 20 s to generate a uniform foamable intermediate. The mixture was poured into an open mold for free-rise foaming. After volume stabilization, the sample was demolded following a 3-min rest and thermally cured at 200 °C for 2 h to yield the PI foam.

Step 3: Preparation of ASPI foam. Shear-stiffening micropowder was dispersed in ethanol and applied onto the PI foam at a mass ratio of 15:1. The impregnated foam was vacuum-dried at 80 °C for 24 h to produce the final adhesive stiffened polyimide (ASPI) foam composite.

S2. Figure

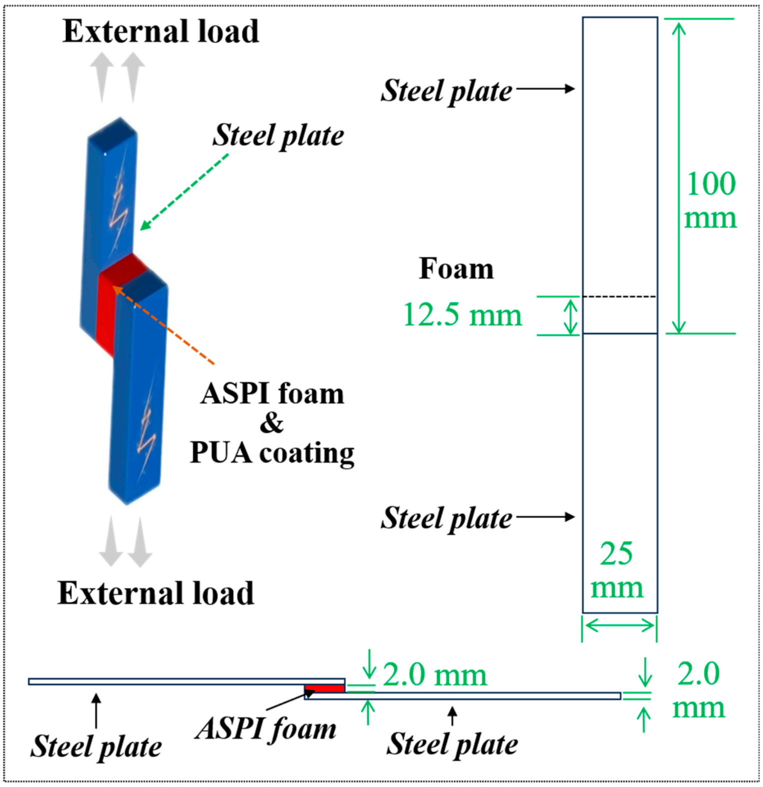

Figure S1. The schematic diagram for adhesive strength test.
